# Functional Surfaces via Laser Processing in Nickel Acetate Solution

**DOI:** 10.3390/ma16083087

**Published:** 2023-04-13

**Authors:** Elena Manuela Stanciu, Alexandru Pascu, Cătălin Croitoru, Ionut Claudiu Roată, Daniel Cristea, Mircea Horia Tierean, Iosif Hulka, Ioana Mădălina Petre, Julia Claudia Mirza Rosca

**Affiliations:** 1 Materials Engineering and Welding Department, Transilvania University of Brasov, Eroilor 29 Str., 500036 Brasov, Romania; 2 Materials Science Department, Transilvania University of Brasov, Eroilor 29 Str., 500036 Brasov, Romania; 3 Renewable Energy Research Institute—ICER, Politehnica University Timisoara, 138 Gavril Musicescu Street, 300774 Timisoara, Romania; 4 Department of Industrial Engineering and Management, Faculty of Technological Engineering and Industrial Management, Transilvania University of Brasov, 500036 Brasov, Romania; 5 Department of Mechanical Engineering, University of Las Palmas de Gran Canaria (ULPGC), 35017 Las Palmas de Gran Canaria, Spain

**Keywords:** laser treatment, microalloying, diffusion, microstructure, liquid media, surface hardening

## Abstract

This study presents a novel laser processing technique in a liquid media to enhance the surface mechanical properties of a material, by thermal impact and micro-alloying at the subsurface level. An aqueous solution of nickel acetate (15% wt.) was used as liquid media for laser processing of C45E steel. A pulsed laser TRUMPH Truepulse 556 coupled to a PRECITEC 200 mm focal length optical system, manipulated by a robotic arm, was employed for the under-liquid micro-processing. The study’s novelty lies in the diffusion of nickel in the C45E steel samples, resulting from the addition of nickel acetate to the liquid media. Micro-alloying and phase transformation were achieved up to a 30 µm depth from the surface. The laser micro-processed surface morphology was analysed using optical and scanning electron microscopy. Energy dispersive spectroscopy and X-ray diffraction were used to determine the chemical composition and structural development, respectively. The microstructure refinement was observed, along with the development of nickel-rich compounds at the subsurface level, contributing to an improvement of the micro and nanoscale hardness and elastic modulus (230 GPa). The laser-treated surface exhibited an enhancement of microhardness from 250 to 660 HV_0.03_ and an improvement of more than 50% in corrosion rate.

## 1. Introduction

Recently, significant attention was devoted towards finding efficient means of performance improvement of metallic parts, through surface modification with energy- and cost-efficient methods. To improve the hardness, wear behaviour and corrosion resistance of metallic materials, various processes can be employed, such as bulk or surface thermal or thermochemical treatments or coatings deposition. Among the conventional methods, laser processing has long been implemented as an efficient means of micro-processing and/or microalloying of metallic surfaces. The laser processing of materials in liquid media is a promising technique which can significantly improve the surface properties of metallic materials, due to a combination of advantages of a liquid media in terms of absorption, confinement, wave transmitting, vaporization and liquid convection effects [1]. Nowadays, in order to overcome the unwanted thermal effects produced by laser processing (i.e., reducing the heat-affected zones), numerous studies are oriented to underwater laser welding [2,3,4], cutting [5,6], cladding [7,8] and peening in liquid media [9]. Laser processing in water is a challenging process due to the numerous parameters and factors that can influence an already complex technology.

The formation of a plasma plume in liquid media is achieved once the laser irradiance threshold is reached in a focal volume. Once the threshold is reached, a rapid heating of the liquid in the near vicinity occurs, followed by its explosive expansion and the emission of a shock wave. The expansion of the heated volume further results in the formation of a cavitation bubble (maximum threshold radius of 1 to 3 mm) [10]. Further expansion of the cavitation bubbles will lead to their collapse generating a shock wave. According to Zhong [11,12], when the bubble collapses, a thin superheated region reaching temperatures up to 9700 °C is formed around the bubble surface [12]. This superheated region is responsible for the thermal and chemical effects in materials processing, while the shock wave is responsible for the mechanical processing.

Considering the physical processes involved, the laser processing in liquid media can be divided into two main categories: CW and long pulse laser processing (ms, µs) and ns/ps/fs ultrashort laser processing. The main differences consist in the pulse interaction with the material, respectively, heat conduction into the material, melting, vaporization and plasma formation for long laser pulses.

For ultrashort laser pulses (ps, fs) the interaction with the material is characterized by a neglected thermal interaction and a direct solid–vapour transition due to short timescale followed by high intensity plasma formation [13,14]. In liquids, these phenomena produce kinetic energy in the form of shock waves and extra pressure (plasma-induced pressure) and formation of cavitation bubbles with the effect/benefit of mechanically influencing the irradiated material [15,16,17]. Moreover, plasma formation in liquid can lead to a thermochemical reaction favourable to nanoparticle synthesis being a promising alternative to chemical methods.

No plasma or low intensity plasma will be produced at power densities below 10^6^–10^7^ W/cm^2^ and only an explosive boiling effect will occur producing a vapour-generated pressure.

If the liquid has a high absorbance of the laser wavelength, then a thermocavitation phenomenon can be obtained as demonstrated by J. P. Padilla-Martinez et al. [18,19,20].

As can be found in the literature, laser processing in liquid with low power continuous or long pulsed lasers are currently used for welding, heat treatment or cutting, the process being based on the continuous heating of the target material. Nikolic et al. [21] used an adaptive neuro fuzzy inference system to improve the forecast of the water-jet assisted underwater laser cutting parameters. Stainless steel up to 60 mm in thickness was cut by laser in water using a continuous ytterbium fibre laser according to Shin et al. [22]. Very good results in terms of efficiency and of secondary waste with applicability for dismantling nuclear facilities have been obtained. In a recent study, Xiangru Feng [23] reported the addition of titanium and the deposition of a zinc-based coating onto a nickel aluminium bronze substrate in an underwater laser cladding set-up. The samples revealed improved corrosion resistance and grain-boundary refinement of the coating microstructure. In a complementary study, the same research group [24] presented the interactions between laser, water and the substrate, highlighting the benefits of underwater laser cladding with titanium rods.

As mentioned above, by using ultra short laser pulses a shock wave can be produced in a liquid media to improve the fatigue strength and resistance of a material (LAL—laser ablation in liquid, LPL—laser peening in liquid). Compared to other laser processing methods, laser peening is a cold process. Hoppious et al. [25] were able to increase the hardness of AISI 316 stainless steel by femtosecond laser ablation in water.

Wang et al. [26] used water and aluminium foil as the confinement layer for laser peening of 5083 Al alloy, thus obtaining an increased hardness by creating a controlled micro-dimple array on the processed surface. Furthermore, using high-pressure shock waves, Wang et al. [27] successfully improved the microhardness and wear resistance of NiTi shape memory alloy by laser shock peening in distilled water.

Compared to laser shock peening, laser heat treatment (hardening) is a processing technique that allows reaching high heating and cooling rates of the processed materials. The conventional laser heat treatment (LHT) uses air (or argon) quenching of the treated area, and the heating stage can be performed with continuous or pulsed lasers. Lapounge et al. [28] carried out extensive research on the behaviour of martensitic and dual-phase steel, subjected to a wide range of temperatures during the laser heat treatment process. According to the authors, the ratio between the spot size of the laser and the thickness of material is of significant importance, being directly linked to the surface properties.

The complexity of the liquid media laser processing phenomena is evident from some of the studies found in the literature. The finite element analysis was used by Telrandhe [29] to predict the depth of the heat-treated zone for a moving laser heat source. Sehyeok and Hyungson [30] used a 3D thermal simulation to predict the hardness distribution as the function of the temperature gradient during laser heat treatment of H13 high speed steel tools with a 94.4% accuracy. Moreover, Hyungson Ki [31] proposed a process map for the laser heat treatment of AISI 1035 and 1020 by correlating the mathematical models with the experimental results. Furthermore, the optimisation of laser processing parameters towards a uniform hardening of the surface was reported [32,33,34]. However, the laser heat treatment/alloying in a liquid media is not understood at full potential.

The main objective of this paper was to obtain an improvement in the surface properties of mild steel (hardness, wear and corrosion resistance), by millisecond-pulsed laser processing in an aqueous nickel acetate solution. The phenomena locally involved at the surface of the sample submerged in the nickel acetate solution include (i) nickel deposition on the sample’s surface due to lower reduction potential of Ni^2+^, (ii) nickel diffusion into a thin layer of material and (iii) in situ quenching of the material (martensite formation). While there are relatively few studies involved in laser processing in liquids, performed with uniform heating, cooling or surface ablation of the material avoiding unwanted solid phase transformations in mind, there are no studies related to the processing of metal alloys in aqueous salt environments. These environments could allow for a more uniform processing of the material, while also providing a diffusional rich environment for nickel. Nickel improves the hardness of the material through the solid solution hardening effect, increases the elastic moduli of the material and decreases the corrosion rate via the surface passivation effect.

The goal was to determine the surface structural changes during processing in aqueous environment without ablation, melting or, at least, with steady-state surface melting (i.e., melting occurring without splashing of molten material, oxide bifilms or inclusions). Therefore, the parameters related to the laser (pulse energy, pulse duration and frequency) and the liquid height were varied and the surface of the material was analysed for ablation/(uneven)melting traces after 60 consecutive pulses. The effect of micro-alloying and phase transformation on the morphology was investigated by optical and scanning electron microscopy. The chemical composition and structural development were analysed by energy dispersive spectroscopy and X-ray diffraction, respectively. The influence of laser heat treatment on the surface toughening mechanism are aimed at providing some useful guidance for researchers in the field of surface treatment.

## 2. Experimental Details

The substrate used in this study was C45E steel (EN 10083-2) with the nominal composition presented in Table 1. The C45E is a medium carbon steel that can be hardened by quenching and tempering, being characterised by a very good machinability and modest weldability. It is commonly used in the automotive industry for manufacturing of shafts, axes and gears.

Circular specimens with dimension of 24 × 4 mm (diameter × thickness) were cut from a cylindrical bar of C45E steel. Prior to the laser processing, the surfaces of the specimens were mechanically polished to a final roughness of Ra = 0.4 µm.

**Figure 1:**
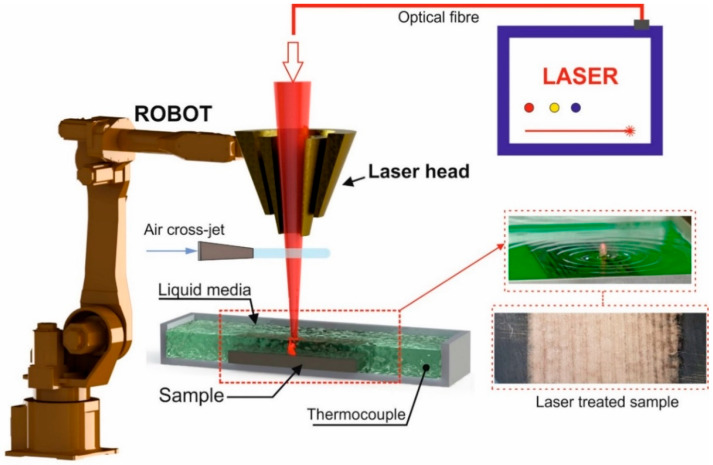
Principle of the laser heat treatment in the nickel acetate liquid media.

The liquid media used for the laser processing was an aqueous solution of nickel acetate (15% wt., close to its solubility limit at room temperature). Nickel (II) acetate tetrahydrate Ni(CH_3_COO)_2_·4H_2_O was purchased from the Chemical Company, Iasi, Romania, and had a purity >99.5% wt. The steel samples were processed using a Nd:YAG TRUMPH Truepulse 556 laser system (λ_max_ = 1064 nm) and a welding optical module made by PRECITEC (WC50) at 200 mm focal length. A powerful cross jet of compressed air was used to protect the optical system against the splashes and vapours. Figure 1 shows the experimental setup and images of the liquid surface and steel sample captured during the laser processing.

Usually, laser irradiance values above 10^10^ W/cm^2^ determine the formation of plasma plumes (laser-induced breakdown) in distilled water for a wavelength of 1064 nm [35]. These irradiance values are uncommon in microsecond or millisecond-pulsed NIR lasers, but this threshold value can be greatly reduced (with up to 50% or more) in the presence of “impurities” (in this case, nickel and acetate ions generated by dissociation of the salt), due to a more facile reaching of the critical free electron density required for multiphoton absorption and avalanche ionization processes. The threshold irradiance values can be reduced with a factor of up to 7 for tap water, compared to distilled water [36].

Through experimental optimization, it was determined that an energy of 380 mJ/pulse at 480 Hz repetition rate is the optimal set-up for obtaining minimal surface ablation (a 1–2 µm surface scattering was inevitable due to plasma formation). This setup is able to produce an irradiance value of 2.3 × 10^5^ W/cm^2^, enough to initiate plasma formation in the presence of dissolved nickel acetate and iron oxide.

Another important factor is liquid level, the relative position of the cavitation bubble related to the free surface of the solution (air-liquid interface), respectively. If the bubble expands and reaches the free surface before collapsing, it will burst, creating a liquid jet.

**Figure 2:**
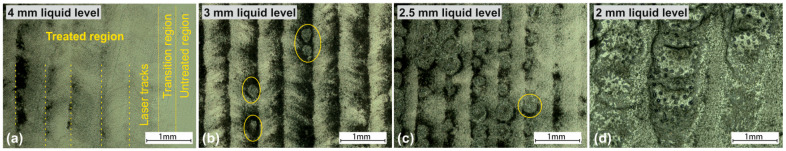
Optical microscopy images, showing the effect of the laser lines at different nickel acetate solution levels; (**a**) treated region without observable surface damage, (**b**) surface with melted spots, (**c**) surface with multiple melted spots and (**d**) severe melting of the surface, laser power 500 W, 0.38 J laser energy, pulse duration 0.7 ms, 480 Hz repetition rate.

If the bubble reaches its maximum radius and collapses before touching the liquid–air interface, a shock wave will be generated towards the steel sample. In this situation, and only if the bubble reaches the critical radius near the surface, the liquid surface is raised forming a liquid dome. A low layer of liquid (1–2 mm) will produce an instable processing with liquid jets and splashes generated by the explosive bubble protruding into the liquid–air interface. An almost “dry” processing situation of the sample is formed, with a significant heat affected zone on the sample surface with visible melted spots (Figure 2b,c) or severe melting (Figure 2d).

A higher liquid level (i.e., 5 to 6 mm) prevents the cavitation bubble from contacting the free surface and stabilizing the process, but too much of the laser power will be dissipated.

After several preliminary tests, it was concluded that the optimum situation is represented by a nickel acetate solution level height of 4 mm above the surface of the sample (Figure 2a). The processing speed was set to 8 mm/s, this value being the threshold below which the surface melting occurs (more than 60 laser pulses per mm^2^ will melt the surface due to the low thermal conductivity of the liquid).

The absorption coefficient of water at 1064 nm is 60 m^−1^ [10,37], which means that around 30% of the pulse energy will be absorbed in the 4 mm water layer. Compared with water, the aqueous solution of nickel acetate (15% wt.) has a higher absorption coefficient of the NIR domain laser beam. All water-soluble nickel salts present a broad absorption maximum in the 850–1200 nm domain, which overlaps with the laser-emitting wavelength (1064 nm) [38]. Moreover, when the liquid is heated and combined with the wave propagation effect, foaming of the liquid media could occur. This phenomenon was mitigated by using a higher pulse repetition rate, which ensured the dissipation of the foam near the processed area.

The liquid media laser processing consisted of 25 partially overlapped lines on the surface of the workpiece and were manufactured with the parameters presented in Table 2.

The experiments were repeated until 10 identical samples were obtained, in order to confirm the repeatability and stability of the process. A new volume of nickel acetate aqueous solution was used each time for the laser processing. The temperature of the liquid media was kept below 50 °C.

The laser processed samples were prepared for microstructural analysis by standard methods of cutting, polishing and chemical etching (Nital reagent—2% concentration).

A scanning electron microscope (Quanta FEG 250, FEI, Hillsboro, OR, USA) was used to assess the material morphology, phase distribution and other structural features. An energy dispersive X-ray spectroscope (EDX with Apollo SSD: detector, EDAX Inc. Mahwah, NJ, USA) was employed to determine the elemental analysis. The 3D surface profile of the samples was analysed by a Hirox KH-8700 digital microscope.

The crystallinity and the phases were determined using a Bruker D8 Discover X-ray Diffractometer (CuK_α1_ = 1.5406 Å), locked-couple technique, step size 0.02, scan speed 3 s/step and 2θ range from 20 to 80°. The overall microhardness of the laser treated sample was analysed using a MICROMET-5124VD Buehler microhardness tester, capable of applying loads from 0.5 up to 2000 gf. Five indentations per sample were made, with the following parameters: 0.5 gf applied load and 10 s dwell time (to minimize the creep effect). The results were averaged and the standard deviation was calculated.

Additionally, the mechanical response at the subsurface level on the laser processed sample and reference sample was measured by instrumented indentation, using an NHT2 nanoindenter module from CSM Instruments/Anton Paar (Buchs, Switzerland), equipped with a three-sided pyramidal diamond Berkovich tip (radius = 100 nm). In order to quantify the depth of the structurally altered material, several indentation loads were employed (from 1 to 500 mN), which translated into several penetration depths. For each load a matrix of minimum 10 indentations was performed, in order to minimize the standard deviation. The indentation protocol was the following: approach speed 2000 nm/min, loading period 30 s, dwell time 5 s (to minimize the creep effect) and unloading period 30 s. The loading/unloading curves were processed using the Oliver and Pharr model. The result of interest was the variation of the indentation hardness and elastic modulus as function of the penetration depth.

The corrosion rate of the laser processed samples was tested using an SP-150 potentiostat/galvanostat and EC-Lab software. A conventional 3-electrodes electrochemical cell was used with the samples as working electrode (laser treated and reference), a platinum grid as counter electrode and a saturated calomel Hg/Hg_2_Cl_2_, KCl sat. as reference electrode. A solution of 9% NaCl was used as electrolyte and the tested area was 10 × 10 mm. The samples were immersed for 1 h in saline solution before the test in order to obtain a steady open circuit potential.

## 3. Results and Discussions

The principle underlying the use of nickel acetate as the precursor for surface modification relies on the Ni^2+^ as mixed basic Ni (II) acetate reduction on the surface of the sample, composed of Feα, due to the highest reduction potential for Ni^2+^/Ni^0^ (E^0^ = −0.25 V) compared to Fe^2+^/Fe^0^ (E^0^ = −0.44 V). The localized increasing in temperature near the surface of the sample determines a further increase in this reduction potential.

Studies regarding the thermal behaviour of nickel acetate indicate the formation of basic Ni (II) acetate non-stoichiometric mixtures (xNi(CH_3_COO)_2_·yNi(OH_2_)) or even NiO [39]. The acetate anion could also be decomposed to formate ions and finally CO and/or CO_2_ due to the localized heating. The CO formed during acetate decomposition could aid in Ni^2+^ reduction to the metallic state [28]. The chemical reactions (Equation (1)) occurring in this surface modification process are presented below [28].

(1) $$\begin{array}{l} {\mathrm{Ni}}^{2+}{+\mathrm{Fe}}^{0}\overset{}{\to }{\mathrm{Ni}}^{0}{+\mathrm{Fe}}^{2+}\\ \mathrm{NiO}+\mathrm{CO}\overset{}{\to }{\mathrm{Ni}}^{0}{+\mathrm{CO}}_{2} \end{array}$$

During the laser processing in liquid, the decomposition of the nickel acetate could influence the convection heating or plasma formation and could act as nucleation sites for the formation of boiling bubbles [18,19,20]. Boiling bubbles can also be formed by the low intensity plasma plume (generated by ablating the surface (Fe and/or deposited Ni)). A part of the laser energy could also be converted into mechanical energy [10,37]. These combined processes could influence the surface morphology of the material; however, the exact laser–liquid–material interaction is beyond the scope of this investigation.

**Figure 3:**
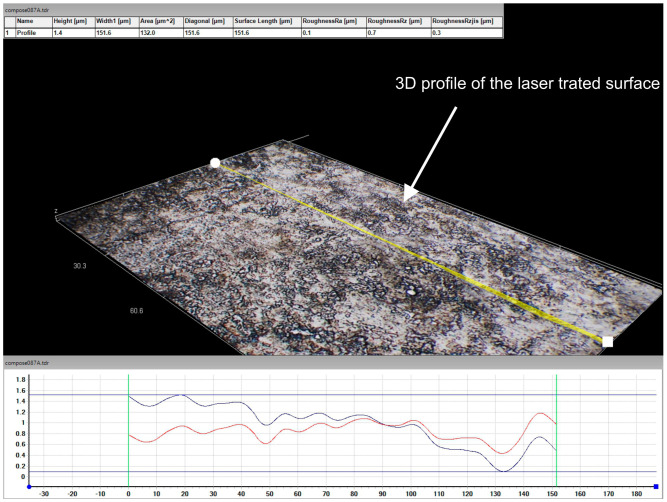
Three-dimensional scanning and profile measurement of the surface after the laser processing (Hyrox 3D digital microscope).

The surface topography profiles of the liquid-processed steel samples indicate Rz roughness values of 0.7 µm (Figure 3), only marginally higher than that for the mechanically polished substrates (0.4 µm), which indicates that (i) a “steady-state” deposition of Ni has taken place and that (ii) melting is most likely to occur (if any) only in localised areas.

**Figure 4:**
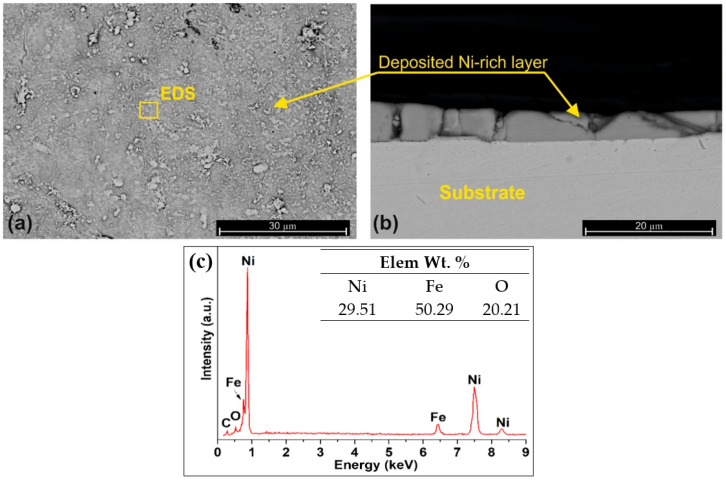
SEM surface (**a**) and cross-section (**b**) of the laser-processed sample at different magnifications; (**c**) EDS analysis of the sample surface.

The as-obtained surface and cross-section morphology of the laser-treated samples reveal the presence of a 0.2–3 µm Ni-rich layer (Figure 4a,b). The EDS elemental analysis shows that the oxygen content of the layer stoichiometrically almost corresponds to NiO, “anchored” to the substrate with inclusion of Fe. Since this layer seemed weakly-bonded to the substrate it was removed by soft polishing.

### 3.1. Microstructure and Phase Identification

**Figure 5:**
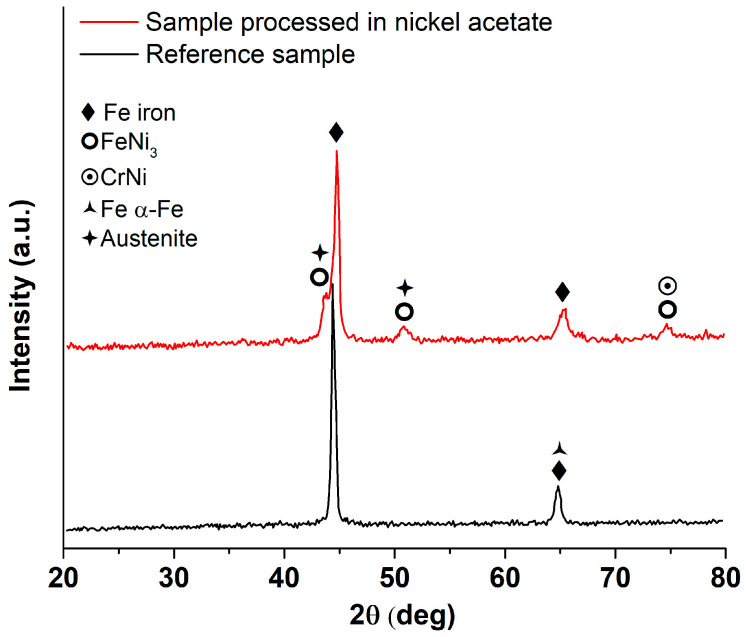
XRD pattern, black line reference sample (without processing) and red line sample processed in nickel acetate.

The possible modifications at the structural level were assessed by X-ray diffraction. Figure 5 represents the diffraction patterns for the liquid media laser processed sample and for the reference sample.

The C45E steel presents two diffraction patterns, which can be ascribed to Fe (44.6°) and α-ferrite (65°). The laser-processed sample presents the peaks ascribed to these phases at higher diffraction angles (44.8° and 65.4°), which could imply a reduction in the α-ferrite unit cell size due to internal stresses. Additionally, new peaks can be seen in the diffraction pattern, which were ascribed to FeNi_3_ and residual Cr, Ni- “alloyed” austenite (γ-Fe). Nickel stabilizes austenite, whose presence can also be explained by strain hardening-stabilizing during martensite formation, since the quenching rate is very high when processing in liquid media.

Broadening of the diffraction peaks ascribed to ferrite (which has a body centred cubic lattice) could signify the distortion of the crystalline lattice to a body centred tetragonal one characteristic of martensite, due to the Bain strain occurring during quenching.

The millisecond pulsed laser delivers a high energy density in each pulse and the heat must be rapidly dissipated into the bulk of the material. The liquid media ensures a direct quenching with high cooling rates as the work piece is kept below 50 ℃. However, the XRD analysis validates the expected microalloying phenomena due to the diffusion of nickel from the nickel acetate solution, mostly due to the formation of the CrNi-type intermetallic compounds. Moreover, if we consider the chemical composition of the C45E steel, the Ni content would not be sufficient for the formation of the FeNi_3_ compound, without a certain degree of Ni supplementation from the liquid solution. These observations support the mechanism presented previously, in relation to the possibility of using nickel acetate as a precursor for the release of Ni atoms during plasma formation.

**Figure 6:**
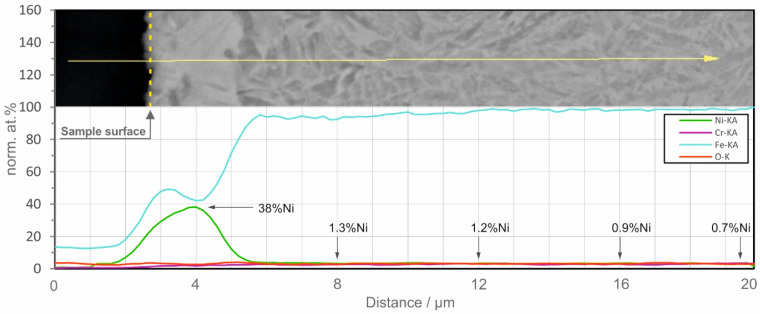
EDS line scan describing the elemental composition throughout the treated region.

The concentration profile dependence for Ni and Fe as a function of distance from the surface shows several irregularities (Figure 6), which can be ascribed to the formation of intermetallic compounds (e.g., FeNi_3_, as evidenced from XRD).

**Figure 7:**
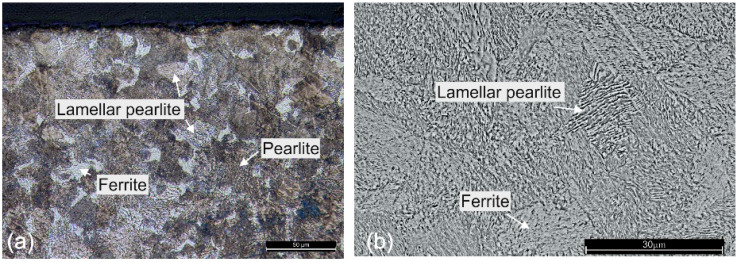
Analyses of the C45 base material, (**a**) optical microscopy and (**b**) SEM analyses highlighting the ferrite pearlite structure.

For the Fe-Ni system, the pre-exponential diffusion coefficient is D_0_ = 6.92 cm^2^/s and the diffusion activation energy is Q = 77.6 kcal/mole, but these constants are valid for pure solids placed in contact with one another. The maximum concentration attained for Ni is ~38 at.% at a 2 µm distance from the top of the workpiece. Relatively similar results were obtained for a classical diffusion experiment at 450 °C for 1200 h [40], but in the present experimental setup, diffusion is expected to occur at a much higher rate, due to the fact that Ni diffusion basically occurs from the liquid phase. Considering a diffusional length of 2 µm, an area of diffusion of 1.130 cm^2^ (considering the diameter of the sample) and an average processing time of 20 s, a diffusion coefficient D = 1.17·10^−8^ cm^2^/s results. In solid-state diffusion experiments, such coefficients are encountered at temperatures above 1390 °C, but an exact comparison is not accurate, due to the fact that diffusion occurs from the Ni^0^, which comes from the Ni^2+^ present in the solution. A detailed structural analysis is presented hereinafter. The base material, in annealed condition, consists of ferrite-pearlite structure with clearly visible coarse lamellar perlite (Figure 7a,b).

**Figure 8:**
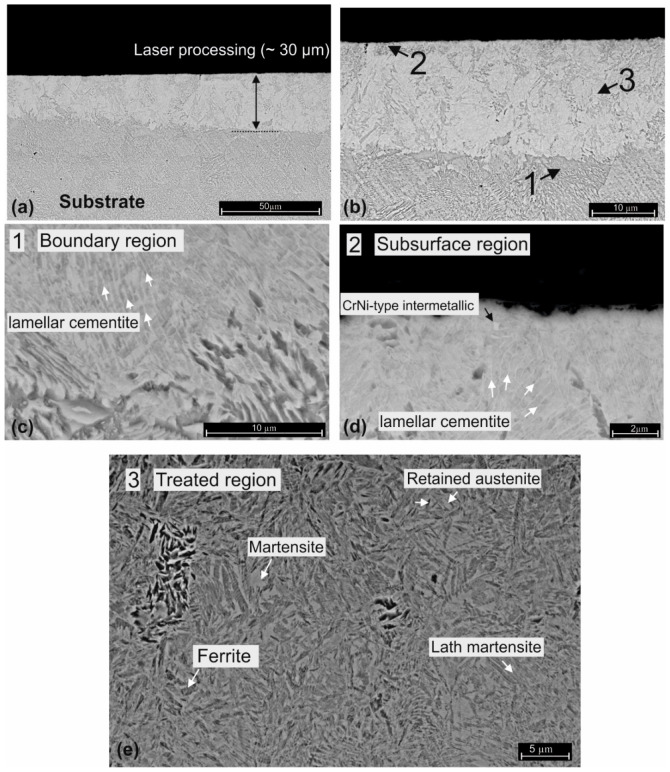
SEM micrographs of the specimen cross section; (**a**,**b**) laser treated region at different magnifications; (**c**) boundary between treated–untreated region; (**d**) subsurface region and (**e**) detail on the treated region (aqua regia etching).

As can be observed in Figure 8a, a phase transformation can be distinguished through a 15–30 µm depth from the surface. This area, visible in Figure 8a,b, is the limit where the ferrite-to-austenite and pearlite-to-austenite transformation process takes place and is also the layer where the maximum diffusion of the nickel occurs. Even if the nickel acetate aqueous solution was a high oxidizing media, decarburizing did not occur, due to the fast heating–cooling process. The white region should not be misinterpreted as surface decarburization. Due to the fast-cooling rate, the complete homogenising of austenite cannot be obtained. The boundary of the heat-treated zone is clearly visible near the thermal processing limit (30 µm depth), where the microstructure consists of some Widmanstätten ferrite structure, retained austenite and lath martensite phase (Figure 8e). Moreover, the incomplete transformation of austenite is denoted by the presence of lamellar cementite phase near the surface (Figure 8c).

### 3.2. Microhardness and Elastic Modulus

**Figure 9:**
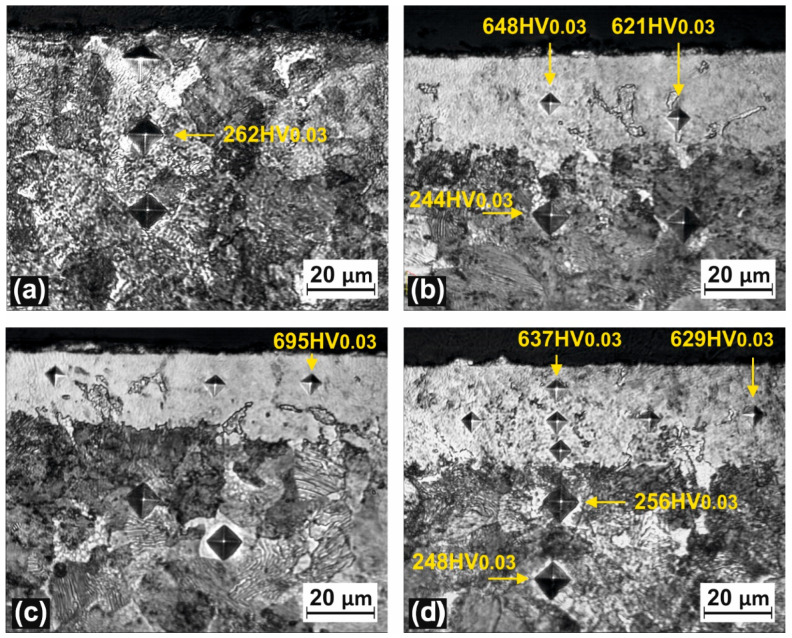
Indentation marks (HV_0.03_) on the cross-section of the untreated sample (**a**) and on laser-treated sample (**b**–**d**).

The purpose of any surface treatment is to improve some characteristics, related to the desired application, such as its hardness, wear resistance, corrosion resistance and so on. The mechanical response of the samples was assessed by microhardness tests and by instrumented indentation. Figure 9 shows the microhardness indentation on the untreated (Figure 9a) and laser treated samples (Figure 9b–d) and Table 3 presents the microhardness profile as the function of depth from the surface.

According to Figure 9 and Table 3, laser processing in liquid media induces a hardening effect. This positive phenomenon is the result of combining the microalloying effect with the rapid pseudothermal processing produced by the pulsed beam. The maximal cross-section microhardness obtained by using the laser processing in nickel acetate solution was 695 Vickers. A high amount of laser energy can be transferred to the surface thanks to the liquid solution that prevents melting of the surface and, at the same time, the liquid solution rapidly cools the treated surface, and this causes the hardness to increase. The microhardness results, corroborated with the SEM and EDS analyses, indicate that the material was micro-alloyed and thermally processed on a depth of 30 µm, followed by a boundary zone of 10–15 µm. According to the EDS analyses, the treated sample exhibits a higher Ni concentration at subsurface level (1–2 µm depth).

**Figure 10:**
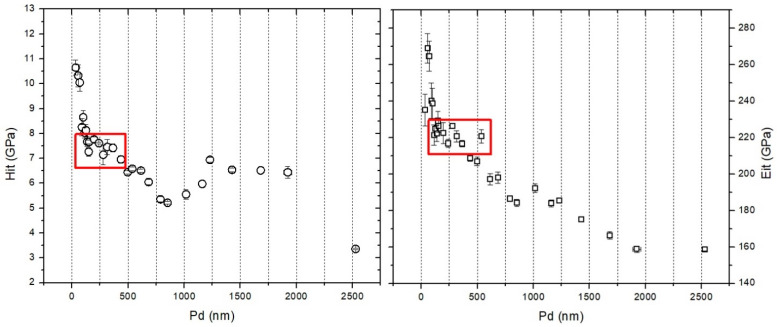
The variation of the hardness and elastic modulus as a function of the penetration depth.

To assess whether this microalloying phenomenon has an influence on the subsurface mechanical properties, instrumented indentation was performed on the laser processed sample, down to a depth of 2500 nm. The variation of the instrumented indentation hardness and elastic modulus as a function of depth is shown in Figure 10.

The formation of Ni-Fe substitutional solutions, as well as Ni-Cr intermetallic compounds determines an increase in hardness, compared to the untreated substrate. The diffusion of Ni in the metallic matrix contributes to the distortion of the crystalline network, with clear effects on the material hardness.

The values of the hardness and elastic modulus obtained for the near surface region (i.e., penetration depth up to 2000 nm) could be related to the Ni-rich subsurface region. Figure 10 reveals that peak indentation hardness (up to 11 GPa ≈ 1100 HV) was obtained near the surface at 100 nm, followed by a stabilization, marked with the red rectangles, which signify that values presented in this stabilized region are characteristic for the region with maximum Ni content (up to 38%, according the EDS line scan presented in Figure 6), hardness between 6 and 8 GPa (≈800 HV) and elastic modulus values between 210 and 230 GPa. Further increasing the load on the indenter, thus reaching higher penetration depths, reveals that decreasing the Ni content significantly affects the elastic modulus, noticeable from the significant drop after 500 nm. The same drop is less pronounced on the evolution of the hardness as function of the penetration depth, after 1500 nm, which could be linked to a work-hardening phenomenon, caused by the diamond indenter.

### 3.3. Electrochemical Corrosion

**Figure 11:**
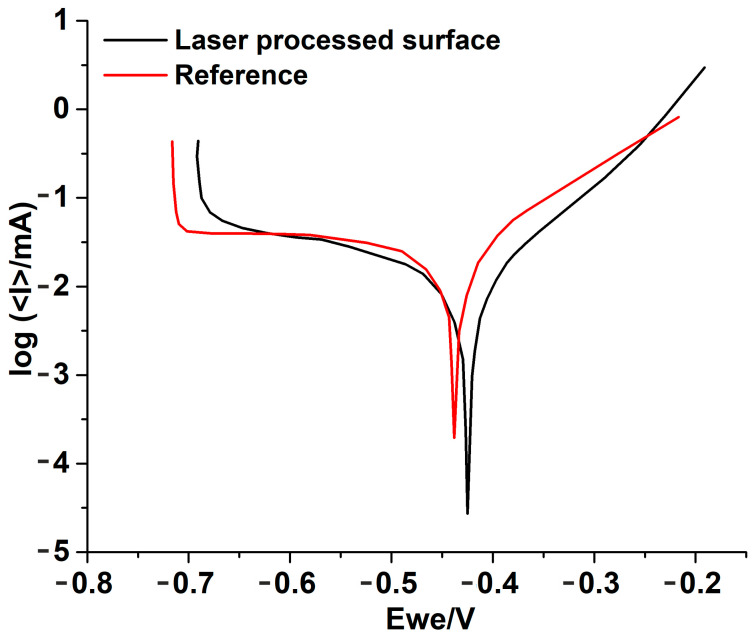
Polarization curves of the reference surface and of the laser processed surface.

In Figure 11 the potentiodynamic polarization curves of the tested samples in 9% NaCl electrolyte are presented. Analysing the presented plots, it can be observed that the laser processed surface has an enhanced corrosion resistance compared to the reference surface.

Table 4 summarizes the corrosion tests’ parameters that generate the polarization curves from Figure 11: Tafel slope constants associated with anodic (ba) and cathodic (bc) processes; E and E_corr_ represent the potential and corrosion potential and i_corr_—corrosion current density.

Improved corrosion resistance was recorded on the laser-treated surface as determined by comparing the polarization curves and the electrochemical data. It is found that corrosion current decreases in the case of the laser processed sample (Table 4) resulting in an improvement of more than 50% for corrosion rate. The improvement in corrosion rate could be due to the presence of nickel, which could passivate the surface of the steel sample when submerged in a saline environment. Furthermore, the presence of the intermetallic compounds and of the martensite phase could have a favourable effect in diminishing the corrosion rate of the material.

## 4. Conclusions

Laser processing in liquid media offers a promising approach to enhance the surface mechanical properties of materials by utilizing thermal impact and micro-alloying at the subsurface level. The use of nickel acetate in laser processing of C45E steel samples led to micro-alloying and phase transformation up to a 30 µm depth from the surface, contributing to an improvement in micro and nanoscale hardness and elastic modulus, and a decrease in the corrosion rate. The laser processed area shows a non-equilibrium martensite structure with residual austenite phase and CrNi and Cr2Ni3 intermetallic compounds formation due to the Ni diffusion from the liquid solution. Both the phase transformation and the microalloying phenomenon contribute to a good mechanical response, with microhardness increasing from 250 up to 660 Vickers units (HV03). The higher subsurface nickel content also leads to a further increase in hardness (8 GPa) and elastic modulus (230 GPa) and improvement of the corrosion behaviour.

This novel laser processing technique opens up opportunities for designing new active liquid media and optimizing process parameters for various materials. This technique can be applied in the biomedical field for the surface modification of implant materials, where increased hardness and corrosion resistance can improve the longevity and performance of the implants. Due to the formation of a plasma plume in liquid media, the technique can also be utilized for micro- and nano-fabrication, as well as in microfluidics and microelectromechanical systems (MEMS) manufacturing.

## Figures and Tables

**Table 1:** Chemical composition of the steel * (C45E|EN 10083-2).

Material	Element wt. (%)								
	C%	Ni%	Cr%	Mn%	Si%	P%	S%	Mo	Fe%
C45 E	0.46	0.12	0.38	0.80	0.09	0.01	0.03	0.06	98.05

* Analysed by SPECTROMAXx arc/spark optical emission spectrometry.

**Table 2:** Laser processing parameters.

Laser Power (W)	Spot Diameter (mm)	Laser Energy (J)	Pulse Duration (ms)	Frequency (Hz)	Speed (mm/s)	Overlap Degree (%)
~184	1	0.38	0.7	480	8	20

**Table 3:** Microhardness profile as function of depth from the surface.

Region	Depth from the Surface (µm)	Microhardness HV_0.03_
Treated region	10	663 ± 9.2
	20	627 ± 3.6
	30	621 ± 4.6
Boundary region	40–50	459 ±11.0
Untreated region	60	259 ± 3.6
	70	248 ± 1.4
	80	246 ± 1.1

**Table 4:** Corrosion test parameters and results.

Operational Parameters	No Processing	Laser Processing
E_corr_	−437.4 mV	−435.5 mV
I_corr_	25.0 µA	11.3 µA
b_c_	669.9 mV	285.1 mV
b_a_	141.9 mV	117.1 mV
Corrosion rate	0.094 mm/year ± 0.006	0.042 mm/year ± 0.003

## Data Availability

Not applicable.
